# Fibrolipoma on upper eyelid in child

**DOI:** 10.3205/oc000040

**Published:** 2016-03-17

**Authors:** Rafael Corredor-Osorio, Nelly Ramos-Pineda, María Eugenia Orellana

**Affiliations:** 1 Instituto Venezolano de Oftalmología, Barquisimeto (Lara), Venezuela; 2 Ocular Pathology, Section Pathology Institute Dr. Jose A. O’Daly, Universidad Central de Venezuela, Caracas, Venezuela

**Keywords:** fibrolipoma, lipoma, benign tumor

## Abstract

An 18-months-old male infant presented with a rapidly growing tumor on the right upper eyelid. Orbital computed tomography (CT) revealed a large, well-circumscribed mass with low density signal in the right upper eyelid. Magnetic resonance images (MRI) showed a lesion of mixed T1-signal intensity and high signal intensity in T2-weighted images. The tumor was treated by simple anterior orbitotomy with excisional biopsy, and the diagnosis of fibrolipoma was made by histopathologic examination. There was no evidence of tumor at the four-year follow-up. Fibrolipoma is one of the rare variant of the lipoma and only four cases have been reported in the orbit including the present case. Except for this case all other cases were reported in adults.

## Introduction

Lipomas are common benign soft tissue neoplasms of mature adipose tissue [1], [2]. Although fat is abundant within the orbit, orbital lipomas are rare [3]. A fibrolipoma is an extremely rare subtype of lipomas from connective tissue tumors composed of mature adipocytes, which are commonly benign [4]. Fibrolipomas belong to the family of fat-containing lesions and are benign tumor characterized by the presence of adipose tissue and abundant amounts of fibrous tissues [3], [4], [5]. They are well-separated from the surrounding tissues and usually occur in adults [5]. Three cases of fibrolipoma of the orbit in adults have been reported. We report another case of the fibrolipoma of the upper eyelid in an 18-months-old male infant.

## Case description

**Figure 1:**
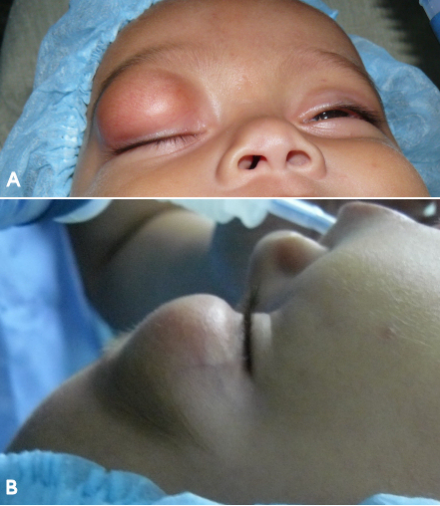
Frontal (A) and lateral (B). Views pre-operatively showing a right upper eyelid mass. Note that overlying skin is not erythematous, edematous or discolored.

**Figure 2:**
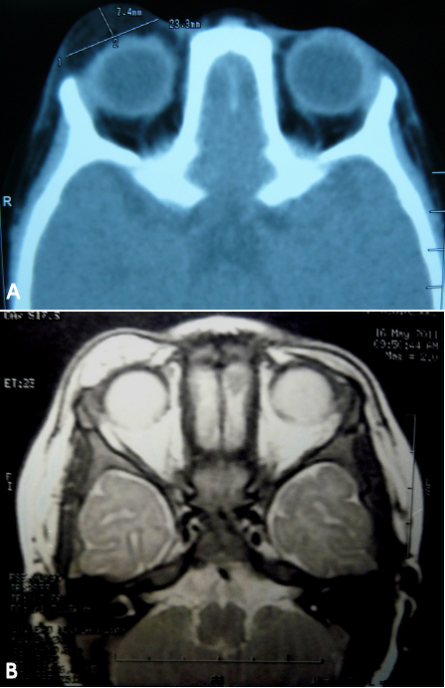
CT images show a 7.4×23.3 mm-sized low density mass in the upper eyelid of the right orbit (A). T2-weighted magnetic resonance imaging (MRI) presenting a high signal intensity (B).

**Figure 3:**
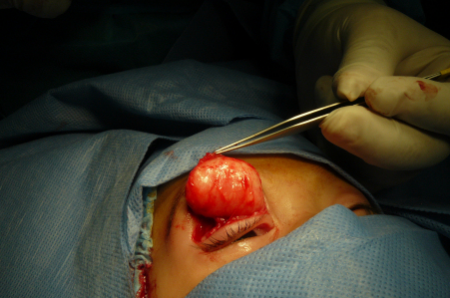
Peroperative photograph shows a yellowish-white soft tissue mass.

**Figure 4:**
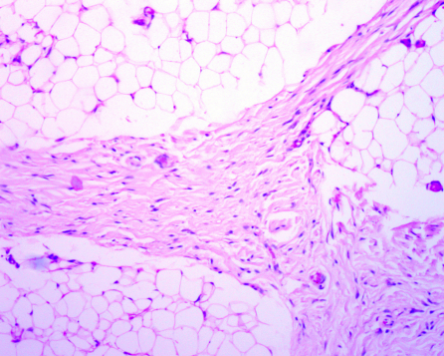
Microscopy showed a lesion composed of mature adipose lobes separated by thick dense connective tissue tracts (H and E, x20).

An 18-months-old male developed a tumor on his right upper eyelid over the course of two months. The tumor became significantly prominent, progressive enlargement over the last month. There was history of trauma to the area. On physical examination he was found to have a very large tumor, approximately 2×2 cm on the right upper eyelid, there appeared to be no discolorations, erythema, or signs of inflammation, it was a firm mass, and nontender to the palpation (Figure 1 (Fig. 1)). The remaining ocular examination was normal. The patient had no systemic manifestations. Orbital CT images demonstrated a large, well-circumscribed mass with a low density signal similar to intraorbital fat on the right upper eyelid. MRI presented a lesion with high signal intensity in T2-weighted images, and the lesion showed a well-marginated soft tissue mass with mixed signal intensity in T1-weighted images (Figure 2 (Fig. 2)). The superior visual field was compromised secondary to mechanical ptosis. The patient underwent an uncomplicated anterior orbitotomy through a superior eyelid crease incision and excision of the tumor (Figure 3 (Fig. 3)). The histological examination of the specimen revealed a lesion composed of mature adipose lobes separated by thick dense connective tissue tracts providing the diagnosis of a fibrolipoma (Figure 4 (Fig. 4)). At follow-up four years postoperatively the cosmetic appearance of the eyelid was very satisfactory and there had been no recurrence of the tumor.

## Discussion

Lipomas are well-circumscribed slow growing, benign tumors composed of mature fat cell grouped in lobules by connective tissue septa [6]. These tumors are very rare in the orbit, accounting for less than 1% of all orbital tumors [7]. The pooled incidence of lipoma was 0.6% in a large orbital tumor series [3] and 1.6% of facial lipomas [4]. Fibrolipoma is a benign tumor that rarely occurs in the orbit, and is classified as a variant of conventional lipoma by the World Health Organization (WHO), because present components of mature adipose cell and bands of dense connective tissue [3], [6], [8]. They occur mostly in adult men [4]. The age of the patient in this case was unusual, because these lesions peak incidence is in the fifth and sixth decade of life [1], [6], and they are rarely observed under the age of 20 years [6]. The clinical features of fibrolipoma vary according to their rate of growth, size and location. Clinically, fibrolipoma usually presents as an asymptomatic, slowly growing mass, firm or soft consistency [4] and well circumscribed [6]. Occurs not only in subcutaneous tissue, but also in various areas such as the oral cavity, trachea, esophagus, parotid gland, spermatic cord [4], [6], larynx, pharynx, colon [6], [9] and nose [4]. According to the literature, it is difficult to value the real incidence of this neoplasm because it appears as painless and slow-growing clinical appearance. In reality, the patient refers to the clinician only when it becomes symptomatic and for functional problems. The size of tumor depends on the location of the lesion. Most of the lesions are less than 3 cm in size, but occasionally lesions become much larger. The consistency of this lesion varies from soft to firm, depending on quantity and distribution of fibrous tissue and depth of the tumor [1], [9]. A characteristic feature is a change in consistency and form of many of these tumors during contraction of involved muscle. The tumor is soft and flat when the muscle is relaxed and becomes firm and more spherical when muscle contracts [2]. MRI is very useful for the diagnosis of all kinds of lipomas. Tumors have high signal intensity on T1-weighted images, with relative decreasing signal on T2-weighted images. A fat suppressed MRI is particularly beneficial for diagnosis. 

Fibrolipomas are more heterogeneous than lipomas on MRI images. On CT images, lipomas can be seen as an uncontrasted hypodense mass [6].

The exact etiology of lipomas and their variants is not well established. An association with genetic aberrations has been described for most types of adipocytic tumor and some trauma mechanisms, such as origin from lipoblastic embryonic cell nest, blunt trauma or chronic intermittent compression, have been associated with the apparition of lipomas [10]. Fatty degeneration, hormonal basis, infection and infarction, metaplasia of muscle cells and chronic irritation are probable representative theories to elucidate the pattern of lipoma [1], [2]. Fibrolipoma has been thought to be congenital to be caused by endocrinal imbalance, to be the product of a degenerated fibromatous tumor, or to arise from maturation of lipoblastomatosis [2]. In our case, the patient had no systemic disease, and he did not have any history of trauma, neither family history of fibrolipoma. Histologically lipomas are classified as a simple lipoma or variant as angiolipoma, chondroid lipoma, myolipoma, spindle cell, pleomorphic lipoma, diffuse lipomatous proliferations (lipomatosis) and hibernoma [2], [6], [8]. Some of these show distinctive clinico-pathological features that are usually discernible only after histological examination [8]. Histologically, when there is a proliferation of components of mature adipose surrounded by dense fibrous cell connective tissue, the tumor is labeled a fibrolipoma [1], [3], [4]. Additionally, fibrolipomas have a higher proliferative activity than the classic variants [6], [9] but no differences in clinical behavior were noticed after surgical treatment [2]. Although fibrolipomas are benign tumors, there are few cases of conversion to liposarcoma in the literature [6]. Fibrolipoma should be differentiated from the other microscopic variants. The angiolipoma consists of an admixture of mature fat and numerous small blood vessels [10], and usually affect male adolescents [11]. Myxoid lipoma microscopally, these lipomas were well-circumscribed and contained adipocytes of variable size and myxoid areas too, exhibits a mucoid background and may be confused with myxoid liposarcoma [11]. The spindle cell lipoma demonstrates a variable amount of uniform appearing spindle cells in conjunction with a more typical lipomatous component. Pleomorphic lipomas are characterized by presence of spindle cells and bizarre hyperchromatic giant cells. Intramuscular lipomas are often more deeply situated and have an infiltrative growth pattern that extends between skeletal muscle bundles [1]. The clinical differential diagnosis considered for the present case includes epidermoid cyst and dermoid cyst. 

Because of the diverse modes of presentation, some other lesions should be considered in the clinical differential diagnosis and these include fibrous histiocytoma, fasciitis nodular and schawannoma. Definitive diagnosis can be based on histological appearance. The treatment of fibrolipoma, including all the histological variants is complete surgical excision. In our case the patient requested the removal of his lesion because of concerns rapidly growing, and the esthetic even so functional aspects resulting from the compression of focal structures. The most common symptom described was rapidly growing painless swelling. Medical management of lipomas includes steroid injections (triamcinolone acetonide) that result in local fat atrophy, thus, shrinking the tumor size. They are best done on lipomas that are less than 2.54 cm in diameter. Average volume of steroid used may range from 1 to 3 ml depending on the size of tumor [12]. In summary, fibrolipoma is one rare variant of the lipoma. In the present case, the treatment of choice was a complete resection of the tumor. Due to the similarity of clinical pictures of all lipomas, the histopathological examination of the excised tissue is the gold standard for diagnosis. The prognosis of fibrolipoma is excellent and recurrence is rare. Fibrolipoma should be included in the differential diagnosis when a localized mass in the eyelid is encountered. Therefore it is essential for ophthalmologists to be familiarized with these lesions. The case presented here adds to the existent 3 cases of fibrolipoma orbit reported in the literature.

## Notes

### Competing interests

The authors declare that they have no competing interests.
